# Optical Hyperpolarization of ^1^H and ^19^F Spins in Organic Liquids

**DOI:** 10.1021/acs.jpclett.6c02456

**Published:** 2026-08-29

**Authors:** Federico De Biasi, Lorenzo Franco, Marco Ruzzi, Gabriele Stevanato

**Affiliations:** Department of Chemical Sciences, 9308 University of Padova, via Marzolo 1 35131 Padova, Italy

## Abstract

Optical dynamic nuclear polarization (oDNP) in liquids offers a microwave-free route to NMR signal enhancement but has so far relied on a limited number of dye–radical systems with restricted photostability, typically requiring high-power laser sources and sample flow to mitigate photobleaching of the dye. Here, we investigate porphyrin–BDPA pairs for oDNP in organic solvents using illumination from an incoherent light-emitting diode, providing ^1^H enhancement factors up to –7.5 at 0.35 T and –25 at 0.16 T in bulk solutions. Beyond ^1^H hyperpolarization, we show optical enhancement of ^19^F nuclei in bulk solutions (+5 at 0.37 T), with the positive enhancement indicating a significant scalar contribution to the electron–nuclear interaction. The improved photostability of porphyrins enables one- and two-dimensional oDNP experiments without sample flow, thereby simplifying the experimental implementation of oDNP at low magnetic fields.

## Graphical abstract

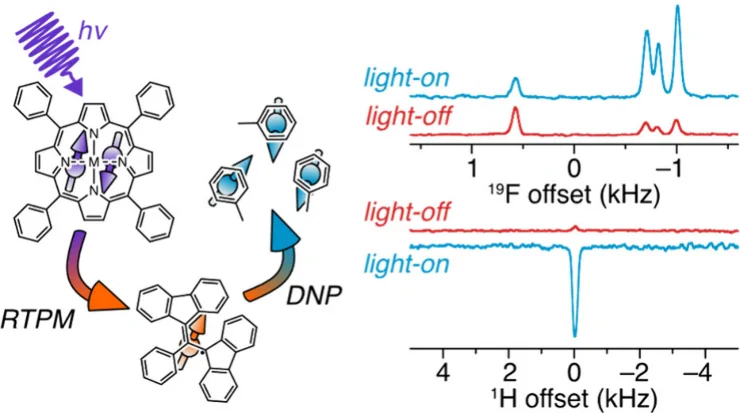

Hyperpolarization methods boost nuclear spin polarization far beyond thermal equilibrium, overcoming the intrinsic sensitivity limits of NMR spectroscopy.^1–4^ Beyond sensitivity enhancement, they accelerate multidimensional experiments and enable the observation of heteronuclei with a high analytical value. Among these, ^19^F NMR is particularly attractive, because of its large chemical shift dispersion and broad relevance in chemistry, materials science, and biomedicine.^5–7^ Dynamic nuclear polarization (DNP) remains the most widely used hyperpolarization approach in solids and liquids, where microwave irradiation is used to transfer electron spin polarization to the nuclei.^8–15,1,16,17^

Optical DNP (oDNP) generates nonequilibrium electron spin polarization using light rather than microwave irradiation.^18–24^ In solution, this occurs when a photoexcited triplet chromophore interacts with a stable radical, a process described by the radical–triplet pair mechanism (RTPM).^25–30^ The stochastic triplet–radical encounters generate transient electron spin hyperpolarization that can be transferred to nearby nuclear spins via the Overhauser effect.^31,32,13^ Despite its conceptual simplicity and high potential, liquid-state oDNP has remained largely limited to ^1^H detection and to a small number of dye–radical systems. The largest reported bulk solvent enhancement (ε = −4), calculated as ε = (*I*_light_ – *I*_dark_)/*I*_dark_, was obtained at 0.34 T for the ^1^H signal of water using Rose Bengal as the triplet sensitizer and 2,2,6,6-tetramethylpiperidin-1-oxyl (TEMPO) as the stable radical, and required irradiation with relatively high-power lasers and flow conditions to mitigate Rose Bengal photobleaching.^19–21^ The negative sign of the enhancement reflected the dominance of dipolar electron–nuclear interactions in the polarization transfer process.

Here, we investigate a class of systems for liquid-state oDNP in organic media based on the stable carbon-centered radical 1,3-bisdiphenylene-2-phenylallyl (BDPA) and either free-base tetraphenylporphyrin (H_2_TPP) or zinc tetraphenylporphyrin (ZnTPP) as the triplet photosensitizer (Figure 1). Because of the improved photostability, the pairs operate without sample flow and can be efficiently illuminated using a subwatt light-emitting diode (LED). This simplifies liquid-state oDNP experiments while achieving bulk toluene ^1^H enhancement factors up to –7.5 at 0.35 T and –25 at 0.16 T. In addition to ^1^H hyperpolarization, optical enhancement of ^19^F nuclei in bulk perfluorotoluene is demonstrated. In contrast to the negative ^1^H enhancements, the positive ^19^F enhancement points toward a significant scalar contribution to the electron–nuclear interaction. The improved photostability also enables two-dimensional NMR acquisition under continuous illumination at 0.37 T, illustrated here by a ^19^F COSY experiment.

**Figure 1:**
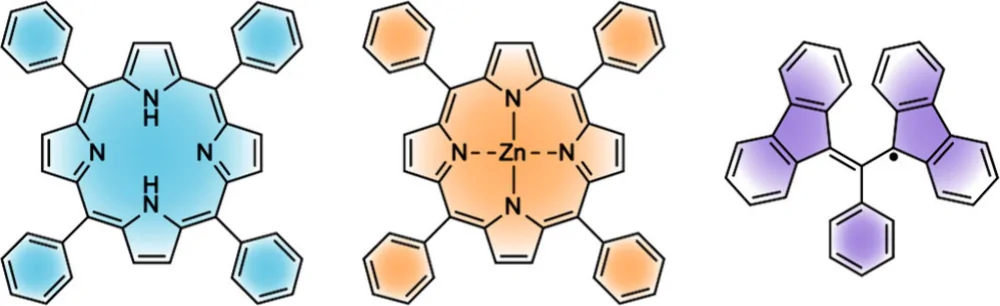
Chemical structures of H_2_TPP (blue), ZnTPP (orange), and BDPA (purple).

Liquid-state ^1^H NMR experiments at 0.16 T and 0.35 T (1617 G and 3513 G) and ^19^F NMR experiments at 0.37 T, 0.56 T, and 0.74 T (3733 G, 5553 G, and 7388 G) were performed using a custom low-field setup. Samples were irradiated at 405 nm using an LED coupled to a 6 mm quartz rod, and the NMR signal was detected with a solenoidal coil enabling *in-situ* illumination. More details on the NMR setup are given in the Supporting Information (SI). All samples were degassed with at least 5 freeze–pump–thaw cycles and flame-sealed under vacuum. During the entire duration of the experiments, the samples were actively cooled using a stream of room-temperature nitrogen gas. Given the geometry of the NMR setup, ≤ 0.4 W of light effectively reaches the sample, as the 0.9 W at the rod exit is spread over ∼ 28 mm^2^, well beyond the < 10 mm^2^ sample cross-section.

The sequence of events leading to nuclear hyperpolarization is sketched in Figure 2. The system starts from H_2_TPP or ZnTPP (singlet ground state, S_0_) and BDPA (doublet ground state, D_0_) at thermal equilibrium. Upon photoexcitation of S_0_ to S_1_, the chromophore triplet state T_1_ is populated via intersystem crossing. Because of stochastic encounters between T_1_ and D_0_, doublet and quartet states (D_1_ and Q_1_) are created due to the distance-dependent radical–triplet exchange coupling (*J*_ex_). *J*_ex_ is modulated by their translational diffusion (an exponential dependence on the radical–triplet distance is assumed). Due to spin-selective decays from the coupled states to the ground state (namely, only the D_1_ → D_0_ + S_0_ transition is spin-allowed) and subsequent distancing of the two species, electron spin hyperpolarization is accumulated on the radical (shown as an emissive polarization in Figure 2b). Finally, the electron spin hyperpolarization is transferred to the nuclear spins (e.g., ^1^H or ^19^F spins) due to the imbalance between the double-quantum and zero-quantum electron–nuclear cross-relaxation rates *W*_2_ and *W*_0_.^31,8,32^ The ∼50-μs-long electron spin longitudinal relaxation time of BDPA in toluene at room temperature (Figure S7) allows efficient accumulation of electron spin polarization via RTPM, maximizing the subsequent Overhauser transfer.

**Figure 2:**
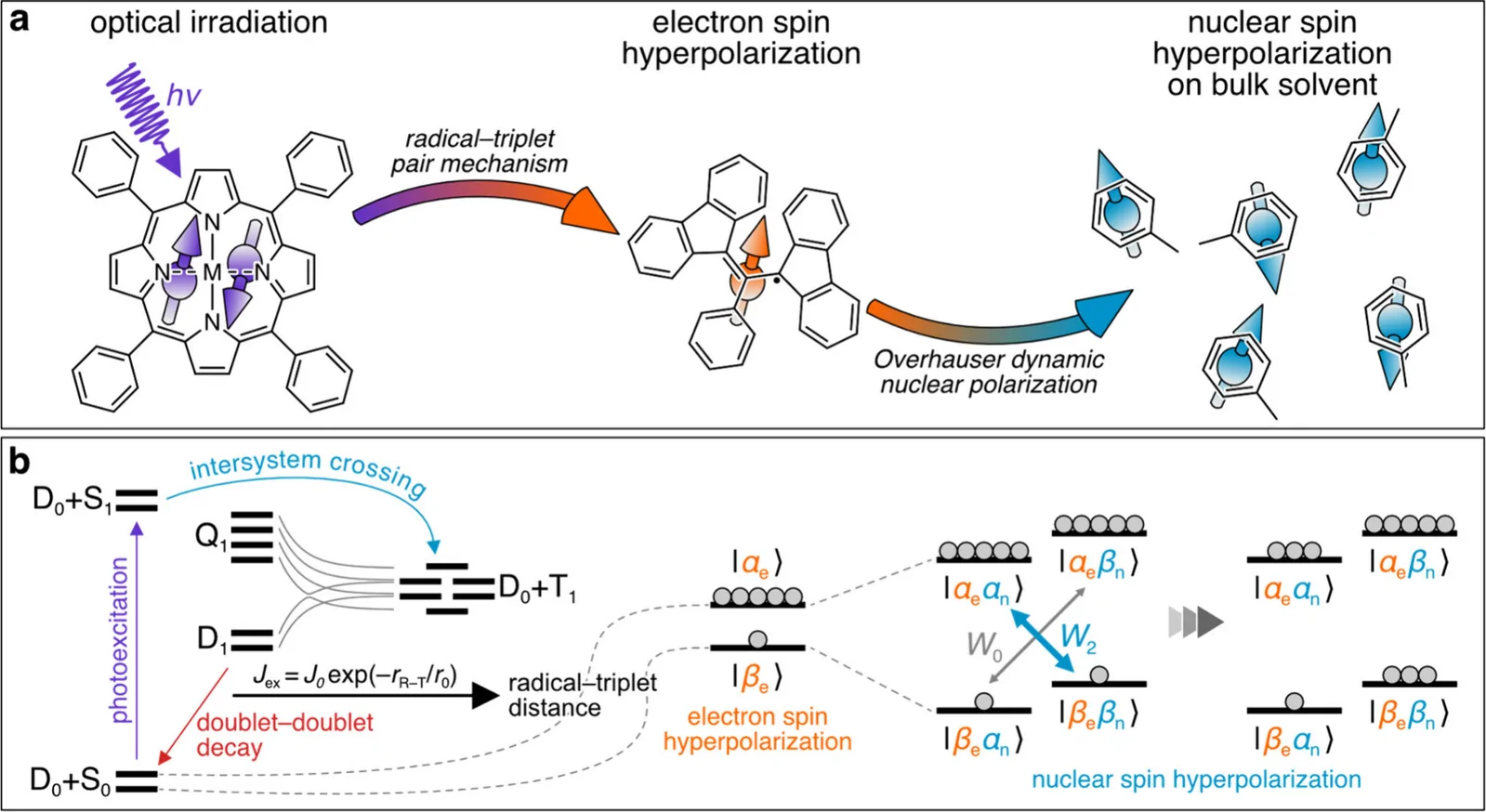
(a) Graphical summary of microwave-free, light-induced nuclear spin hyperpolarization in liquids. A suitable chromophore is photoexcited and RTPM generates electron spin hyperpolarization, which is then transferred to nuclear spins via the Overhauser effect. (b) Energy level diagrams showing the combined action of RTPM and Overhauser DNP to create nuclear spin hyperpolarization.

^1^H oDNP NMR experiments were performed on 0.5 mM BDPA and either 0.2 mM H_2_TPP or 0.2 mM ZnTPP solutions in toluene:toluene-*d*_8_ 1:1 v/v (Figure 3). The magnetic field homogeneity was 10 ppm. The ^1^H NMR spectrum is dominated by the solvent peak, as solvent protons (∼37 M) exceed solute concentrations by more than 3 orders of magnitude. The bulk solvent ^1^H signal enhancement factors have been measured at 0.35 T and 0.16 T for both porphyrin–BDPA pairs at 20 s of light irradiation followed by a 90° pulse-acquire experiment. The enhancements are achieved under mild illumination conditions (≤0.4 W), at room temperature and without sample flow. At 0.35 T, ε = −7 is about twice as large as that reported for Rose Bengal and TEMPO in H_2_O:D_2_O mixtures.^19–21^ We note that, according to the definition of the enhancement factor, the observed ^1^H enhancement at 0.35 T corresponds to a 6-fold increase in signal amplitude, which, under otherwise identical experimental conditions, would reduce the acquisition time required to achieve a given signal-to-noise ratio by a factor of 36.

**Figure 3:**
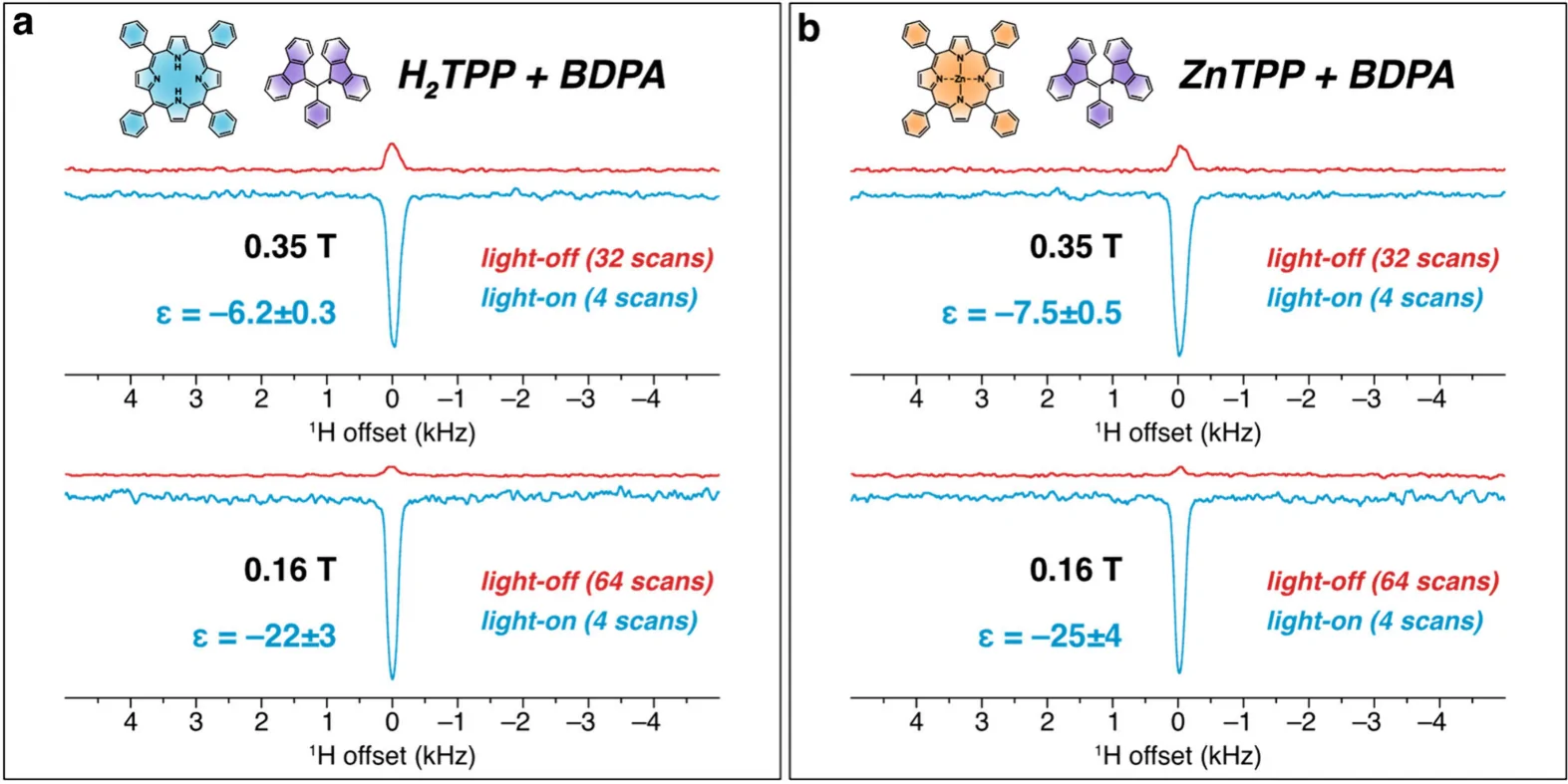
Liquid-state ^1^H NMR spectra (0.35 T and 0.16 T) of 0.5 mM BDPA with either (a) 0.2 mM H_2_TPP or (b) 0.2 mM ZnTPP in toluene:toluene-*d*_8_ 1:1. Spectra were acquired with signal presaturation and a 20 s polarization delay. Light-off (red) and light-on (blue) spectra are scan-normalized. ^1^H signal enhancements are reported in the inset.

Dale and Wedge showed bulk ^1^H enhancements of –4 for the Rose Bengal–TEMPO pair in H_2_O:D_2_O mixtures using a flow setup, 4 s irradiation, and a 2 W laser. The enhancement improved to –6 by the addition of glycerol to increase the solvent viscosity.^19,21^ Liu and co-workers showed that fullerene–nitroxide covalent dyads possess improved photostability over the Rose Bengal–TEMPO pair, though bulk solvent ^1^H enhancements remained only 0.5 at 0.34 T.^33^ More recently, Yabuki and co-workers examined porphyrin–trityl pairs in toluene, reporting bulk ^1^H oDNP enhancements below unity.^29^

The 4-fold increase in ^1^H signal enhancement, from –7 to –25, when halving the external magnetic field *B*_0_ from 0.35 T to 0.16 T arises from two independent contributions: the 2-fold decrease in thermal nuclear spin polarization at 0.16 T and the 1/*B*_0_ scaling of the electron spin hyperpolarization accumulated via RTPM.^34^ Together, these effects yield the observed 4-fold increase in oDNP enhancement at 0.16 T, corresponding to a 2-fold rise in molar nuclear polarization.

At 0.35 T, nearly identical buildup times (∼15 s) are observed in the dark and under illumination, indicating that the enhancement is essentially time-independent (Figure 4). Similar results were obtained at 0.16 T (Figure S3). No measurable photodegradation is observed via NMR after ∼270 s of cumulative continuous illumination (Figure S4), while a 10 to 20% decrease of the BDPA EPR signal is observed after 2 h of pulsed laser irradiation (5 ns, 1 mJ, 50 Hz repetition rate) at 532 nm (Figure S5). Furthermore, the ^1^H enhancement increases with optical power over the range accessible with our LED source at either field (Figure S9).

**Figure 4:**
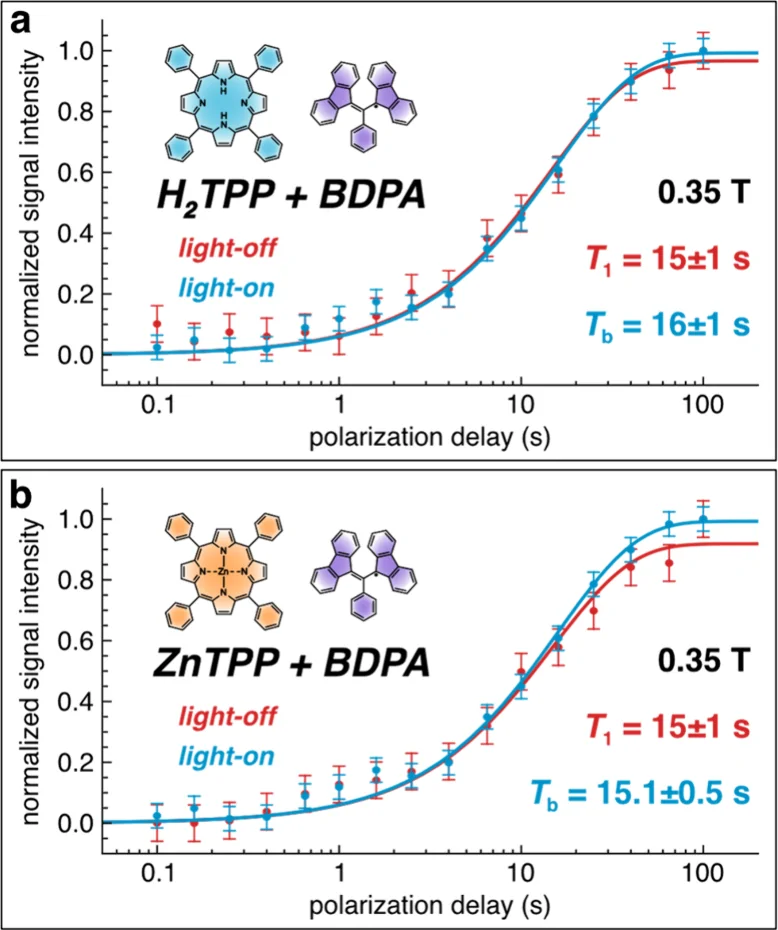
Bulk solvent ^1^H polarization buildups measured at 0.35 T on a sample of 0.5 mM BDPA with either (a) 0.2 mM H_2_TPP or (b) 0.2 mM ZnTPP in toluene:toluene-*d*_8_ 1:1. Buildups were measured in the dark (red, 32 scans per data point) and under light irradiation (blue, 1 scan per data point). Each dataset was fitted to an exponential recovery function *I*(*t*) =*I*_∞_ (1 – exp­(−*t*/*T*_*i*_)) to give the buildup times reported inset (light-off: *T*_1_; light-on: *T*_b_).

We find that H_2_TPP and ZnTPP exhibit similar oDNP performances despite their slightly different zero-field splitting parameters *D*_ZFS_ (1.1 GHz for H_2_TPP and 0.9 GHz for ZnTPP).^35^ This observation contrasts with the quadratic dependence on *D*_ZFS_ predicted by RTPM theory,^34^ suggesting that polarization generation depends on additional factors beyond zero-field splitting alone. Similar photoexcited triplet lifetimes for the two porphyrins in toluene at room temperature (1.5 ms and 1.2 ms for H_2_TPP and ZnTPP, respectively)^35^ support this interpretation.

Time-resolved EPR (TR-EPR) spectroscopy was used to probe the BDPA spin polarization and the response under illumination. The spectra in Figure S6 reveal a strongly emissive hyperpolarization of the BDPA signal after the laser pulse, together with a markedly long-lived character with a decay constant similar to the electron spin *T*_1_ (Figure S7). The emissive polarization of BDPA is consistent with the negative ^1^H enhancements observed here, which relate to a dipolar-dominated Overhauser mechanism between BDPA and the toluene ^1^H spins. Indeed, the Overhauser DNP coupling factor for BDPA and toluene ^1^H spins has been measured at the X-band by Levien and co-workers to be 0.14.^36^ Notably, this coupling factor is below the dipolar limit of 0.5, resulting in suboptimal polarization transfer from the radical.

The restriction of liquid-state oDNP experiments to ^1^H spins has so far limited its spectroscopic scope. To assess whether the approach could be extended beyond ^1^H, we investigated ^19^F, a nucleus of considerable interest, due to its large chemical shift dispersion and widespread applications in chemistry, materials science, and biomedicine.^37^ As shown in Figure 5a, a positive bulk solvent enhancement factor of +5 is observed for the aromatic ^19^F spins in a perfluorotoluene:toluene-*d*_8_ 1:1 v/v mixture under 20 s of light illumination at 0.37 T with 0.5 mM BDPA and 0.2 mM H_2_TPP, demonstrating that liquid-state oDNP can be extended to ^19^F nuclei. Given the negative electron spin polarization of BDPA accumulated via RTPM (Figure S8 for the TR-EPR data in perfluorotoluene), a positive ^19^F enhancement indicates a significant scalar contribution to the Overhauser polarization transfer mechanism. This contrasts with the predominantly dipolar Overhauser transfer observed for toluene ^1^H spins.^39,40^ The scalar ^19^F Overhauser transfer in perfluorotoluene is likely promoted by transient π– π stacking between the electron-deficient ring of perfluorotoluene and the delocalized aromatic spin cloud of BDPA, which brings the ^19^F nuclei into direct contact (i.e., Fermi contact) with the radical spin.^41–43^ The decrease in ^19^F enhancement with increasing magnetic field (3.3 ± 0.4 at 0.56 T and 1.8 ± 0.2 at 0.74 T, Figure S10) is consistent with the 1/*B*_0_ field dependence predicted for RTPM-driven electron spin hyperpolarization.

**Figure 5:**
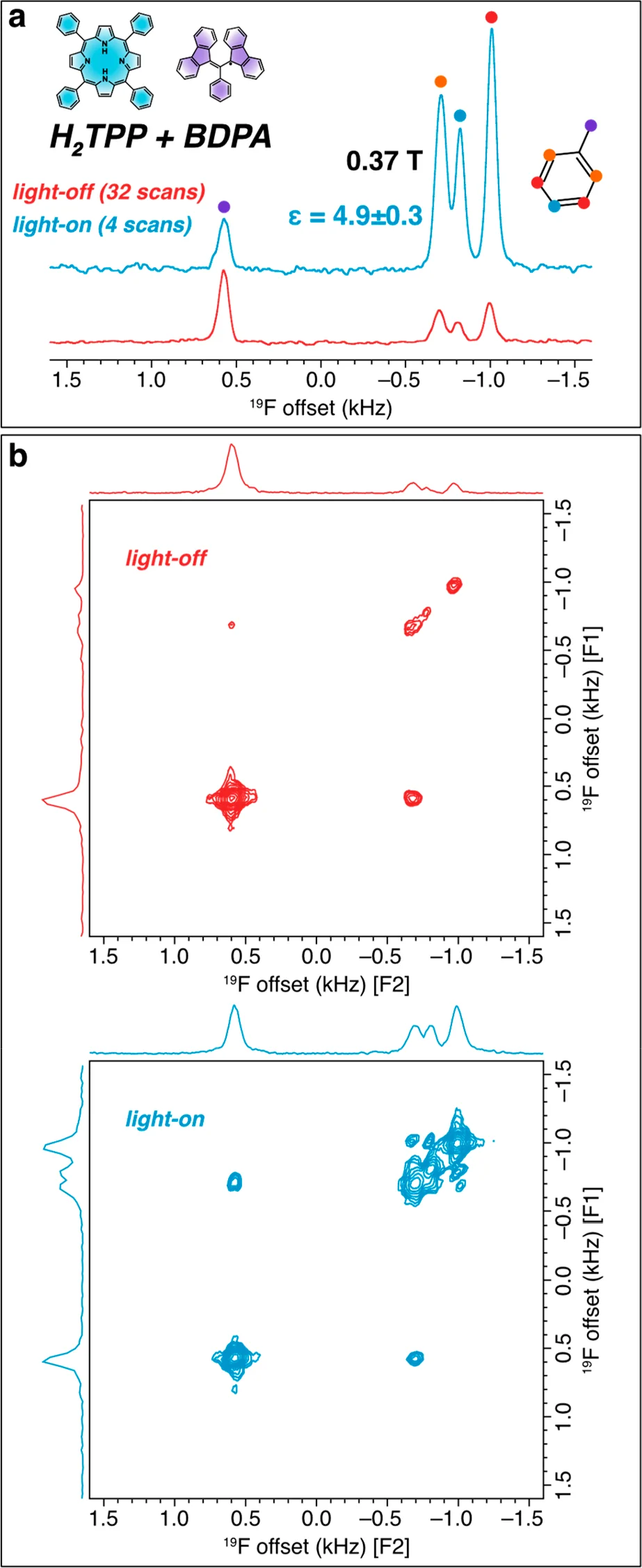
(a) Liquid-state ^19^F NMR spectra of 0.5 mM BDPA and 0.2 mM H_2_TPP in perfluorotoluene:toluene-*d*_8_ 1:1. Spectra were acquired at 0.37 T with signal presaturation and a 20 s polarization delay. (b) 2D ^19^F COSY-90 light-off (red) and light-on (blue) NMR experiments recorded at 0.37 T on the same sample (3 s recycle delay, 4 scans per transient). In the light-on spectrum, all cross-peaks are visible.

The large ^19^F chemical shift dispersion allowed the separation of the CF_3_ and the three aromatic ^19^F resonances in perfluorotoluene even with a field homogeneity of 10 ppm, a feature that makes this nucleus particularly attractive for low-field NMR spectroscopy and imaging.^5,38^ Two-dimensional ^1^H and ^19^F NMR at low magnetic fields have been shown to enable structural elucidation for microwave-induced Overhauser DNP.^44,45^ In the present context, the improved photostability of the H_2_TPP–BDPA pair also enabled two-dimensional oDNP experiments at 0.37 T under continuous illumination, as illustrated here by a ^19^F COSY spectrum (Figure 5b).

Taken together, our results show that porphyrin–BDPA pairs exhibit suitable photophysical properties for liquid-state oDNP experiments. The synthetic versatility of porphyrins, together with the modular choice of radical partners and opportunities to optimize the radical–solvent polarization transfer, offers considerable scope for further development of liquid-state oDNP. Nonetheless, while this work demonstrates oDNP in organic liquids using porphyrin–BDPA systems, extending this approach to aqueous media while preserving photostability and performance is not merely a matter of solubility. Transitioning to water significantly alters the underlying photophysical parameters affecting the chromophore triplet lifetime, the radical spin relaxation rates, and electron nuclear spin interactions.

Our observations also indicate considerable room for improving the NMR enhancements reported in the present study. The BDPA concentration used here was not optimized, and increasing the radical contribution to nuclear relaxation could increase the leakage factor, favoring the Overhauser polarization transfer. Moreover, the strong absorption of porphyrins in the Soret band at 405 nm likely leads to nonuniform photoexcitation across the sample volume, such that the measured bulk enhancements represent a spatial average and may underestimate the polarization levels achieved locally. Higher bulk oDNP enhancements could be obtained through the optimization of radical concentration, chromophore concentration, optical path length, and illumination geometry, together with radical–solvent combinations characterized by larger coupling factors.

In conclusion, compared with previously reported dye–radical systems, the porphyrin–BDPA pairs investigated here operate in organic liquids, under static conditions, require low-power illumination, and enable optical hyperpolarization of both ^1^H and ^19^F nuclei. The combination of long-lived triplet states and slow radical spin relaxation is consistent with efficient accumulation of electron spin polarization via RTPM. The observed decrease in enhancement with increasing magnetic field indicates that low-field polarization generation is the most favorable operating regime for the present systems for both ^1^H and ^19^F nuclei. Nevertheless, this field dependence does not preclude future implementation of low-field optical hyperpolarization with rapid field-transfer strategies, whereby polarization generated at low field is subsequently detected at higher magnetic fields.

## Supplementary Material



## Data Availability

All the raw NMR data associated with the manuscript can be accessed at the following Zenodo link 10.5281/zenodo.20664694 and is available under the CC-BY-4.0 (Creative Commons Attribution 4.0 International) license.

## Notes

Chemicals and preparation; experimental setup; NMR pulse sequence; light-on buildup times at 0.16 T; porphyrins photostability; EPR steady-state (CW) and time-resolved (TR) spectra; power dependence; ^19^F oDNP experiments at 0.56 and 0.74 T; supplemental figures (PDF)
