# Supplementary material for: Optical Hyperpolarization of 1H and 19F Spins in Organic Liquids
Source: J Phys Chem Lett. 2026 Aug 29;17(37):10691–6. doi: 10.1021/acs.jpclett.6c02456 (PMC13592082; doi:10.1021/acs.jpclett.6c02456)
Supplement: Supplementary file 1 [file jz6c02456_si_001.pdf]

SUPPLEMENTARY INFORMATION

Optical hyperpolarization of ^{1}H and ^{19}F spins in organic liquids

Federico De Biasi, Lorenzo Franco, Marco Ruzzi, and Gabriele Stevanato*

Department of Chemical Sciences, University of Padova, via Marzolo 1, 35131 Padova, Italy

1. Chemicals and preparation 2
2. Experimental setup 2
2.1. Custom-made detection coil and tuning and matching box 3
2.2. Light source 3
3. NMR pulse sequence 3
4. Light-on buildup times at 0.16 T 4
5. Porphyrins photostability 4
6. EPR steady-state (CW) and time-resolved (TR) spectra 5
7. Power dependence 7
8. ^{19}F oDNP experiments at 0.56 and 0.74 T 8

1. Chemicals and preparation

The compounds 5,10,15,20-Tetraphenyl-21H,23H-porphine (free-base porphyrin, H_2TPP, CAS: 917-23-7), 5,10,15,20-Tetraphenyl-21H,23H-porphine zinc (zinc porphyrin, ZnTPP, CAS: 14074-80-7) and 1,3-bisdiphenylene-β-phenylallyl (BDPA, CAS: 35585-94-5) were purchased from Sigma-Aldrich and used as received. For the experiments, all compounds were dissolved either in toluene/toluene-d_8 = 1/1 (v/v) or perfluorotoluene/toluene-d_8 = 1/1 (v/v) mixtures. Porphyrin–BDPA solutions were loaded into quartz capillary tubes (2 mm outer diameter, 1 mm inner diameter). The sample height was approximately 5 mm to ensure uniform illumination along the full volume via the 6 mm o.d. quartz rod used as light guide (see experimental setup below). All samples were degassed by at least five freeze–pump–thaw cycles until no further gas evolution was observed upon thawing, and subsequently flame-sealed under vacuum.

2. Experimental setup

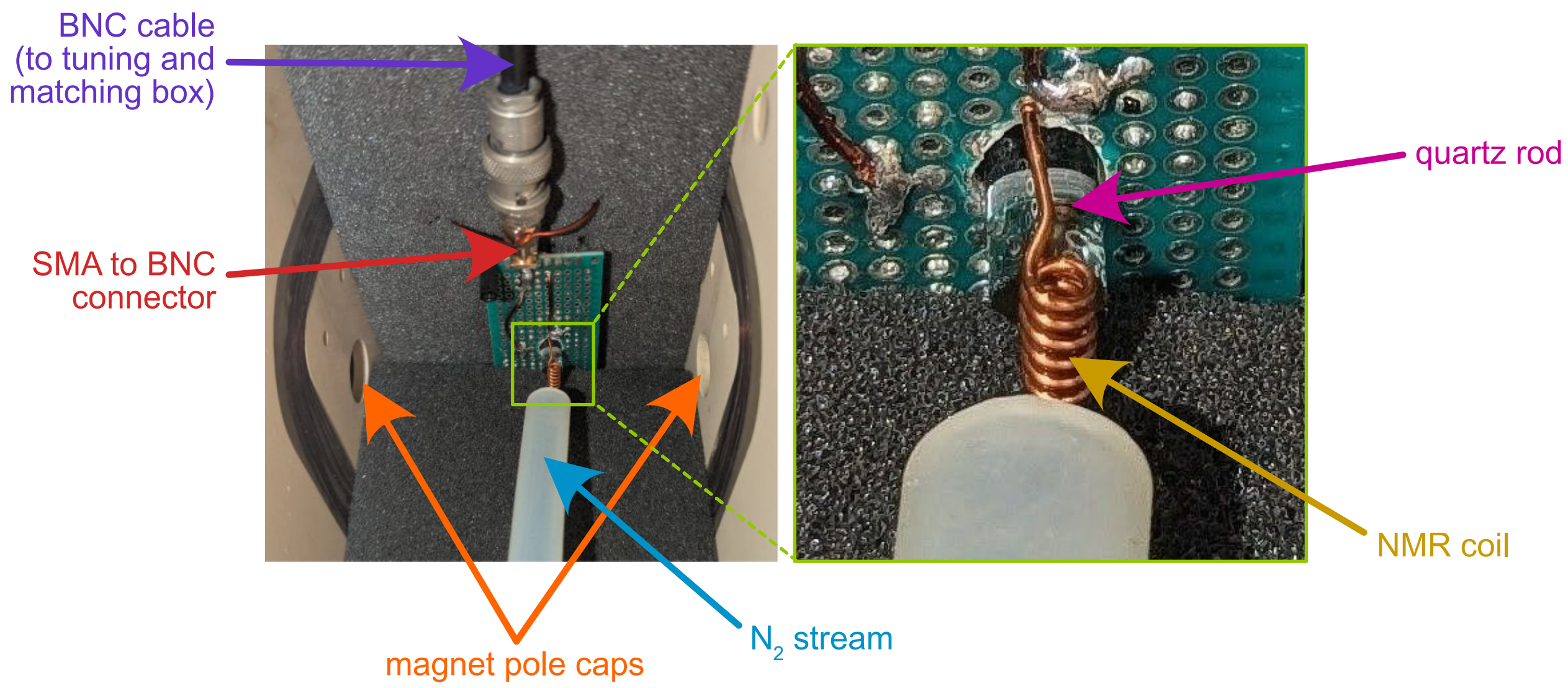

Figure S1. Picture of the experimental setup.

The main magnetic field was generated by a Bruker ER073W electromagnet driven by a Bruker ER083 power supply and regulated by a Bruker ER032M hall field controller, providing a horizontal magnetic field in the range 30–800 mT. Light irradiation of the sample was achieved with a Thorlabs M405LP1 LED source centered at 405 nm, powered by a Thorlabs DC40 LED driver. The LED was coupled to a quartz rod (6 mm o.d.) to guide the light to the sample. The measured optical power at the end of the rod was 0.9 W.

^{1}H and ^{19}F NMR signals were acquired at the field of the electromagnet with a Bruker Avance III NanoBay 400 MHz NMR console using the low-frequency X-nuclei channel, a custom-built solenoidal coil (placed at the end of the rod) and a custom-built tuning and matching box. The spires of the NMR coil were evenly spaced to allow for sample irradiation with visible light. In the pulse program (in Topspin 3.7), a TTL trigger out was inserted to synchronize the light irradiation and the detection. The samples in the capillary tubes (2 mm o.d., 1 mm i.d.), were manually positioned in the NMR detection coil, which was placed at the center of

the electromagnet pole pieces using a non-magnetic support to ensure a stable and reproducible alignment with the light source.

The sample temperature was monitored by placing a thermocouple inside the RF coil region. Under continuous 405 nm illumination and active cooling with a stream of room temperature N_2 gas, switching on the LED resulted in an approximately 4 °C increase in temperature, after which a stable steady-state value was reached within the time resolution of the measurement.

Continuous wave (CW) and time resolved EPR experiments were performed at room temperature on a Bruker Elexys E580 CW/Pulsed spectrometer operating at X-band (9.6 GHz). The time resolved spectra were collected as a series of EPR signal buildup and decay after pulsed laser excitation (Quantel Brilliant Nd:YAG with second harmonic generator, 532 nm, 5 ns/pulse, 1 mJ/pulse, 50 Hz repetition time) recorded at each field position (400 field points) around the resonant lines of BDPA, using the fast digitizer (Specjet) of the spectrometer. The time resolution of the spectrometer is about 100 ns.

2.1. Custom-made detection coil and tuning and matching box

A custom-made solenoidal detection coil (6 turns, evenly spaced, 0.75 mm o.d. copper wire) was mounted on a rigid support (7×3 cm) and connected via an SMA-to-BNC connector to a custom-built tuning and matching network located outside the magnetic field. The circuit was implemented using variable capacitors (C_{tuning} = 160–520 pF, $C_{matching}$ = 0–360 pF) for tuning and impedance matching. The output was connected to the NMR spectrometer via a standard coaxial BNC cable.

2.2. Light source

A light-emitting diode (LED, Thorlabs M405LP1, λ_{center} = 405 nm) driven by a Thorlabs DC40 controller was used for optical excitation. The LED was triggered via TTL lines synchronized with the Bruker console. Light was delivered to the sample through a 6 mm o.d. cylindrical quartz rod. The optical power at the rod exit was measured using an optical power meter (Gentec-eo UNO) and found to be approximately 0.9 W.

3. NMR pulse sequence

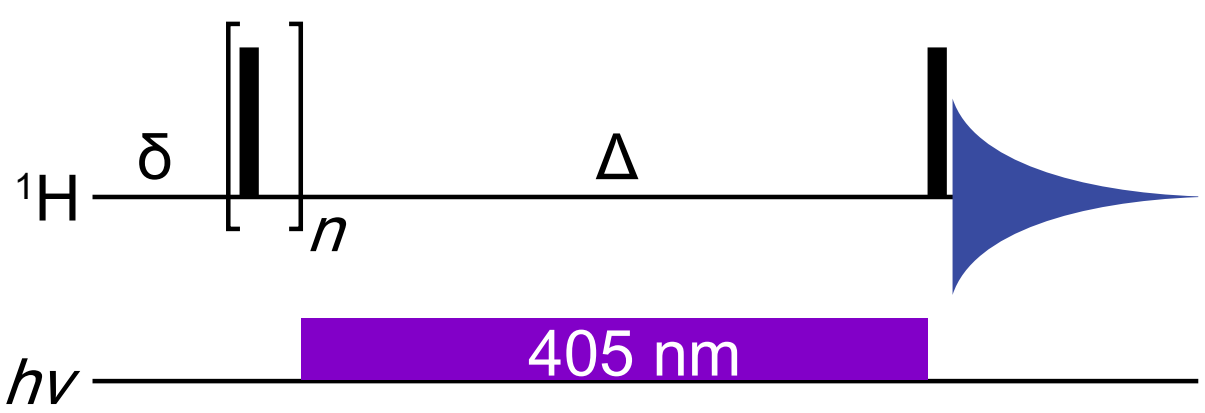

Figure S2. Pulse sequence used for the NMR experiments presented in this work. The initial delay δ is used to allow for the sample to cool down between each scan, if necessary. A train of low-power long RF pulses is then applied to saturate the residual ^{1}H magnetization. The phase of the saturating pulses is constantly alternated between x and y. Light irradiation is performed during the delay Δ, after which a 90° pulse is applied to read the accumulated magnetization. CYCLOPS phase cycling is applied to the last RF pulse and the receiver.

4. Light-on buildup times at 0.16 T

The longitudinal relaxation times with (T_b) and without (T_1) laser irradiation reported in the main text and in Figure S3 were measured using the saturation recovery pulse sequence in Figure S2 by recording the NMR signal as a function of increasing delay Δ. The light-on T_b was also determined at 0.16 T for both samples (Figure S4).

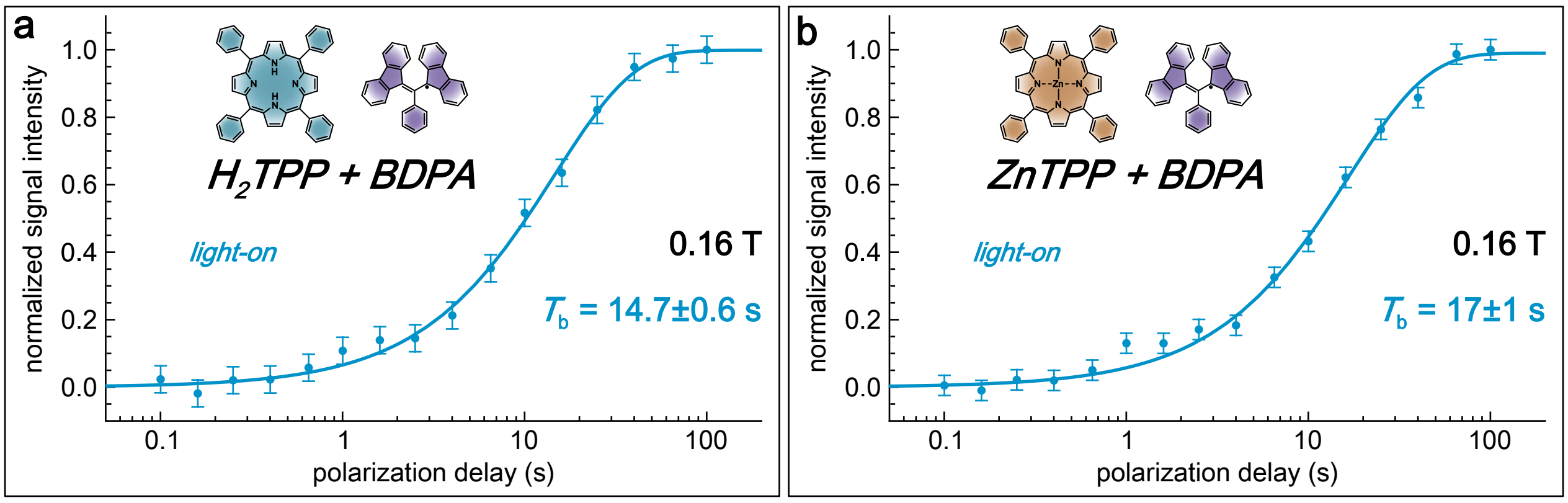

Figure S3. ^{1}H polarization buildups measured at 0.16 T on the bulk solvent signal of a sample of 0.5 mM BDPA with either in a) 0.2 mM H_2TPP or in b) 0.2 mM ZnTPP in toluene/toluene-d_8 = 1/1. Buildups were measured under continuous light irradiation (1 scan per data point). Each dataset was fitted to an exponential recovery function $I(t) = I_\infty(1 - \exp(-t/T_i))$ to give the buildup times reported inset.

5. Porphyrins photostability

Photostability of the H_2TPP–BDPA and ZnTPP–BDPA samples was assessed by comparing light-on NMR spectra acquired before and after the polarization buildup measurements (Figure 4 of the main text, total illumination time of ~270 s at 405 nm). No detectable spectral changes were observed within this timeframe, indicating negligible photodegradation under the experimental conditions.

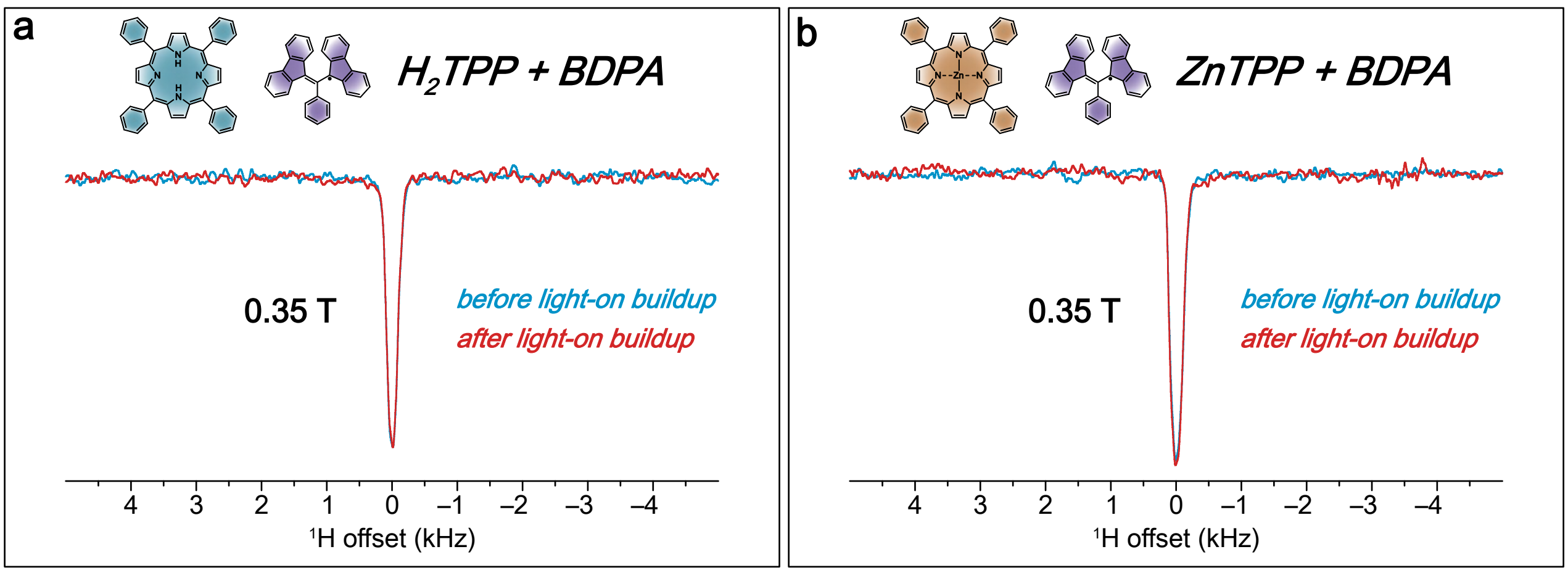

Figure S4. Liquid-state ^{1}H NMR spectra recorded at 0.35 T of 0.5 mM BDPA with either in a) 0.2 mM H_2TPP or b) 0.2 mM ZnTPP in toluene/toluene-d_8 = 1/1. Spectra were acquired using 4 scans each, with signal presaturation and a 20 s polarization delay. Blue and red spectra were acquired before and after the light-on buildup measurements in Figure 4 of the main text, respectively (blue spectra are the same 0.35 T light-on spectra in Figure 3 of the main text).

6. EPR steady-state (CW) and time-resolved (TR) spectra

Continuous wave (CW) EPR spectra were recorded to ascertain the presence and persistence of BDPA radical before and after the laser irradiation of the solutions containing porphyrins and BDPA. EPR samples were prepared in quartz tubes (4 mm o.d., 3 mm i.d.) and degassed with at least three freeze-pump-thaw cycles. Overall, the radical seems to be quite stable even under intense laser photoexcitation of porphyrins. In some cases, the presence of residual oxygen in the solution is causing some bleaching of the radical upon photoexcitation.

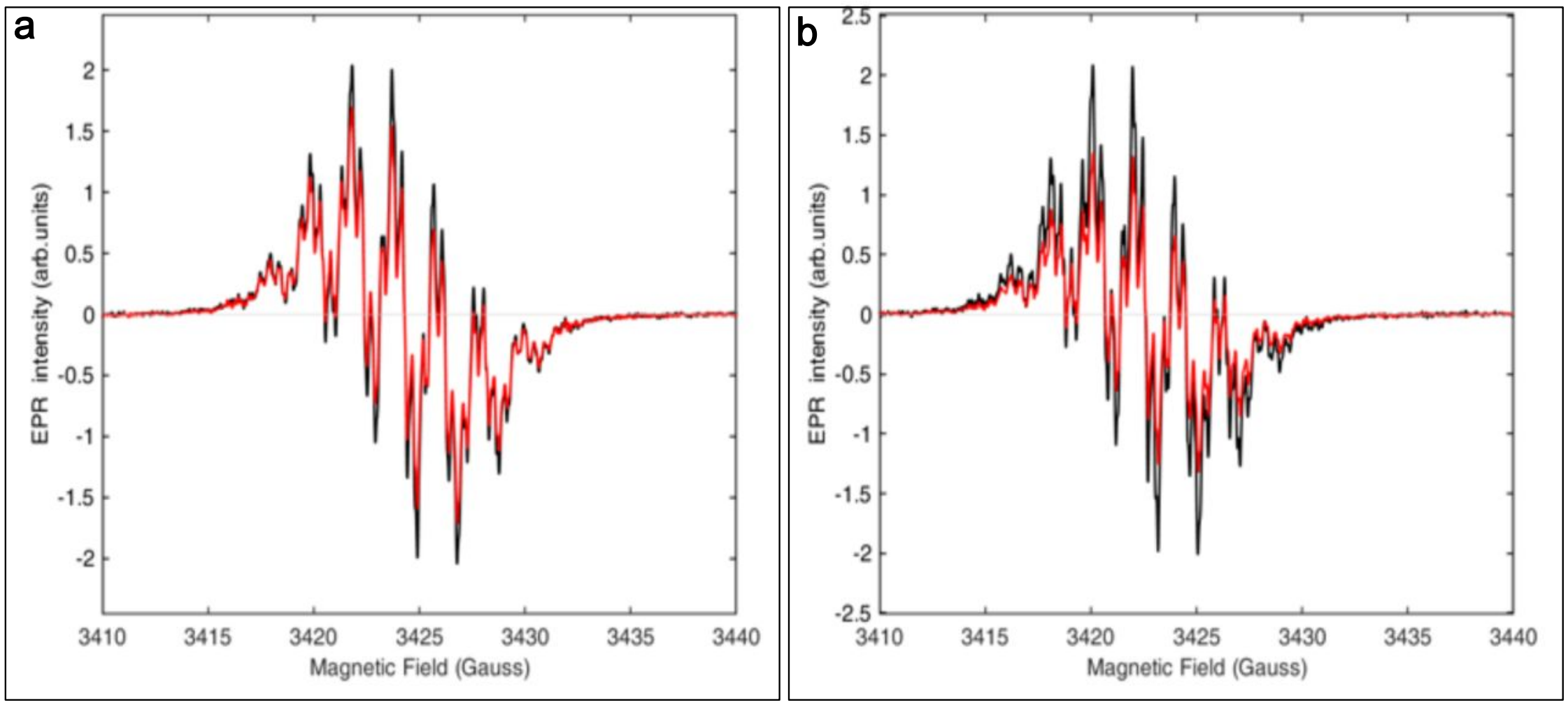

Figure S5. CW EPR spectra of 1 mM BDPA radical recorded at room temperature in toluene/toluene-d_8 = 1/1 solutions in the presence of either in a) 0.2 mM H_2TPP or in b) 0.2 mM ZnTPP. Black lines: spectra before laser irradiation. Red lines: spectra after prolonged (2 hours) pulsed laser irradiation at 532 nm. A 10 to 20% decrease of the BDPA radical is observed after photoexcitation.

Time-Resolved EPR (TR-EPR) spectra were recorded on the H_2TPP or ZnTPP (0.2 mM) and BDPA (1 mM) solutions in toluene/toluene-d_8 = 1/1 at room temperature, using 532 nm laser pulses (5 ns, 1 mJ, 50 Hz repetition rate). For both samples, the TR-EPR spectra correspond to the (integrated) CW EPR spectra and are in emission, with almost identical intensities of the emissive polarization in the two samples. A small variation in the time evolution of the TR-EPR is observed, with the ZnTPP sample having a slightly shorter decay time compared to the H_2TPP sample (45 μs and 60 μs).

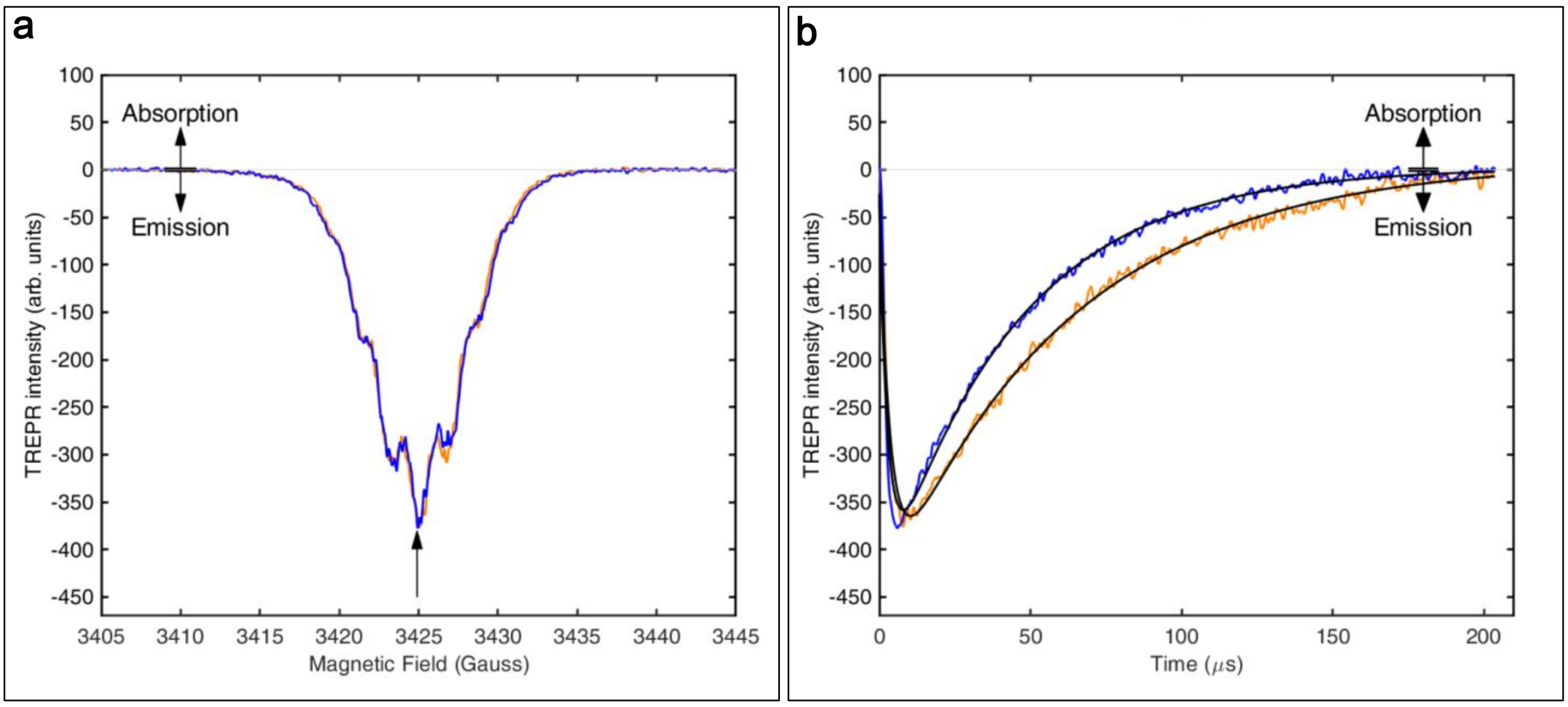

Figure S6. a) Time-resolved EPR (TR-EPR) spectra and b) time evolution of the TR-EPR spectra recorded at the field position indicated by the arrow in panel a. Orange lines: H_2TPP/BDPA sample in toluene/toluene-d_8 1/1. Blue lines: ZnTPP/BDPA sample in toluene/toluene-d_8 1/1. Black lines: exponential best fits of the time evolution of the TR-EPR signal. The time constants of the TR-EPR decays obtained by the exponential fit are 60 and 45 μs for the H_2TPP and ZnTPP samples, respectively. Absorption and Emission indicate a variation of the EPR intensity with respect to the thermal equilibrium intensity before the laser pulse.

The TR-EPR decay is compared with the longitudinal electron spin relaxation time (T_{1e}) of the radical in the 1 mM BDPA and 0.2 mM H_2TPP or ZnTPP toluene solutions. The T_{1e} was measured by pulsed EPR with the spin echo detected inversion recovery sequence. The exponential best fit of the inversion recovery traces, shown in Figure S7, provided the following values: T_{1e} = 55 μs for the H_2TPP sample and T_{1e} = 40 μs for the ZnTPP sample. These values correspond, within experimental uncertainty, to the decay constants of the TR-EPR spectra in Figure S6.

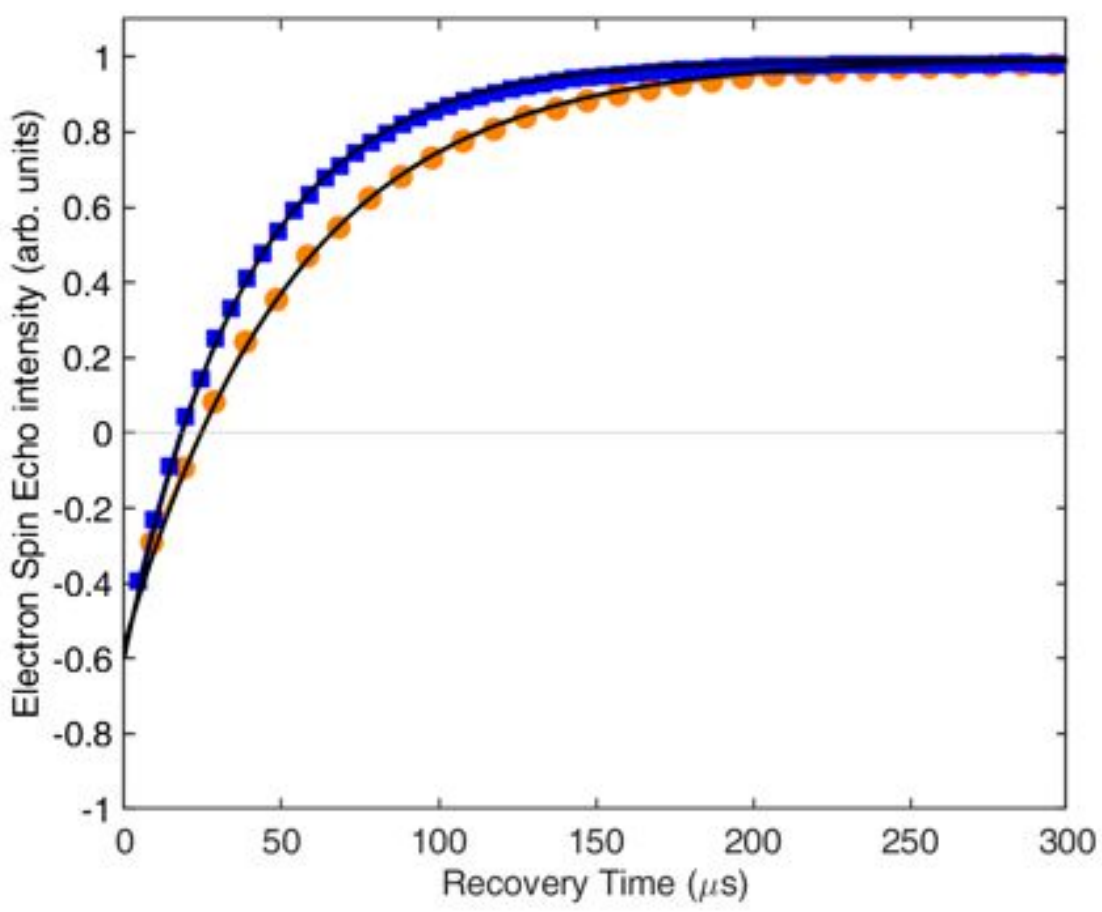

Figure S7. Electron spin T_{1e} relaxation measurements of 1 mM BDPA in the presence of either 0.2 mM H_2TPP (orange circles) or 0.2 mM ZnTPP (blue squares), using the spin-echo detected inversion recovery sequence. Black lines: exponential best fit.

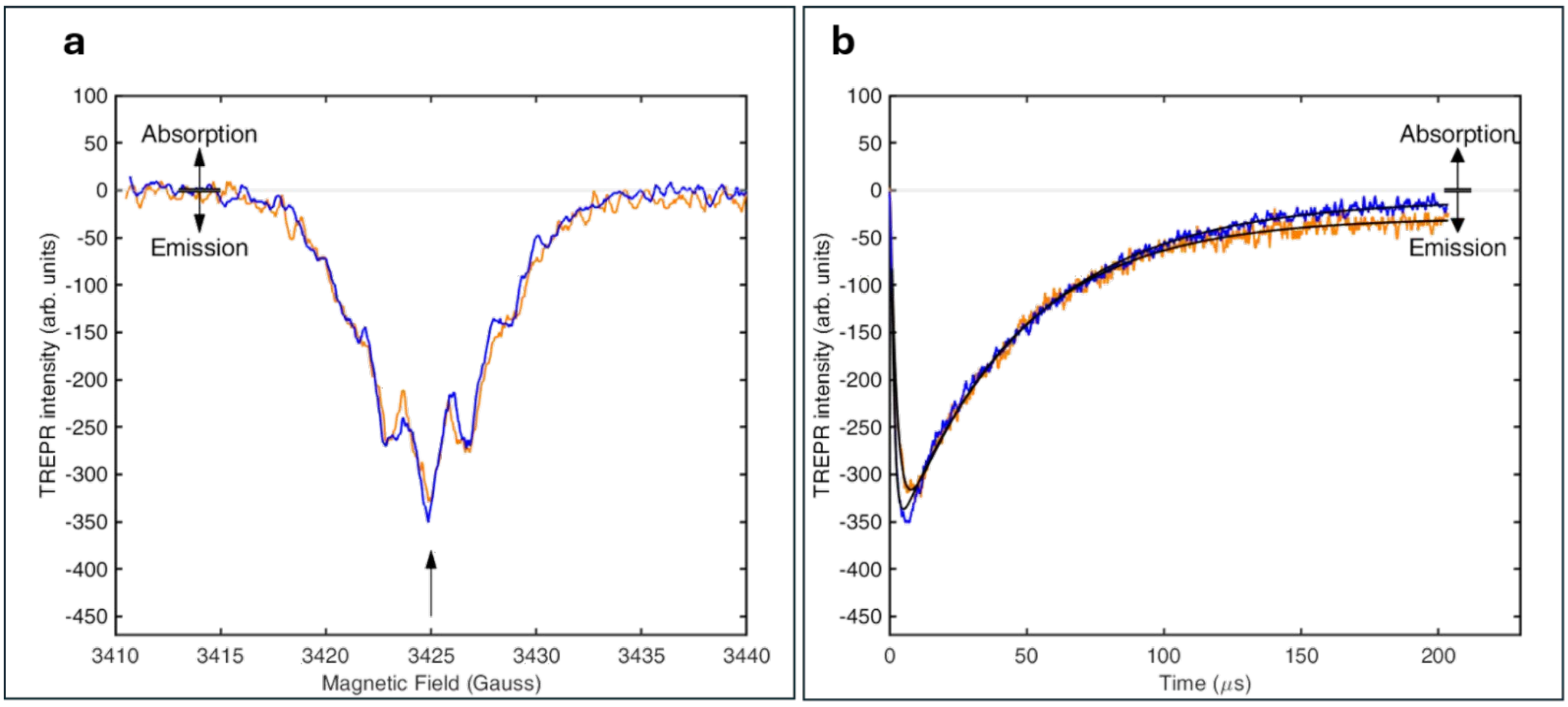

Figure S8. a) Time-resolved EPR (TR-EPR) spectra and b) time evolution of the TR-EPR spectra recorded at the field position indicated by the arrow in panel a. Orange lines: H_2TPP/BDPA sample in perfluorotoluene/toluene-d_8 = 1/1. Blue lines: ZnTPP/BDPA sample in perfluorotoluene/toluene-d_8 = 1/1. Black lines: exponential best fits of the time evolution of the TR-EPR signals. The time constants of the TR-EPR decays obtained by the exponential fit are 49 and 42 μs for the H_2TPP and ZnTPP samples, respectively. Absorption and Emission indicate a variation of the EPR intensity with respect to the thermal equilibrium intensity before the laser pulse.

7. Power dependence

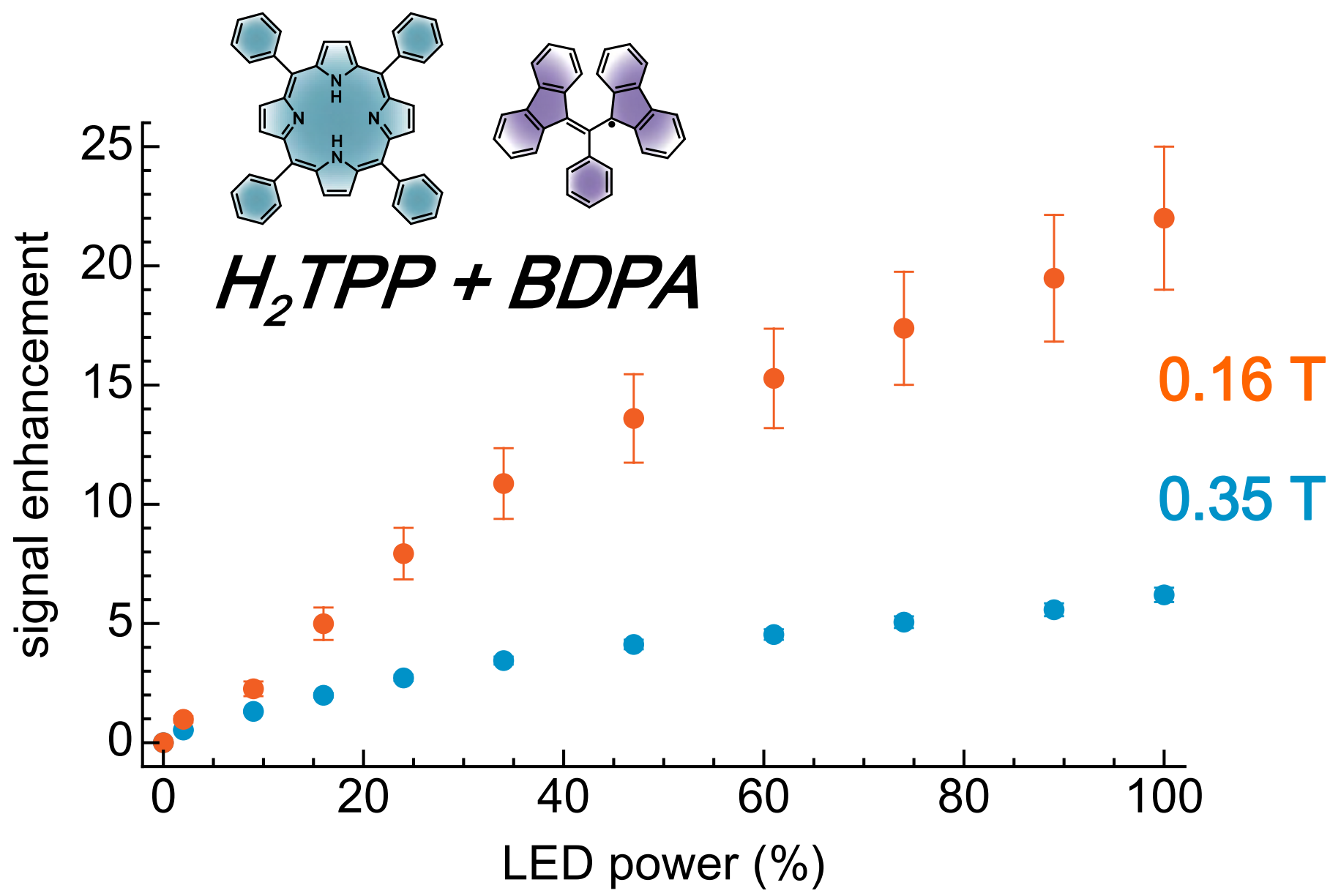

Figure S9. NMR signal enhancement factors for the bulk ^{1}H signal of a sample of 0.5 mM BDPA and 0.2 mM H_2TPP in toluene/toluene-d_8 = 1/1 measured at 0.35 T (blue) and 0.16 T (orange) as a function of the LED power using a 20 s polarization delay. 100% power corresponds to the same illumination conditions of Figures 3 and 4 in the main text. The uncertainty on the enhancement factor has been estimated from the signal-to-noise ratio (snr) of the light-off spectrum as $\delta_\varepsilon = \varepsilon/\mathrm{SNR}_{\mathrm{light-off}}$.

8. ^{19}F oDNP experiments at 0.56 and 0.74 T

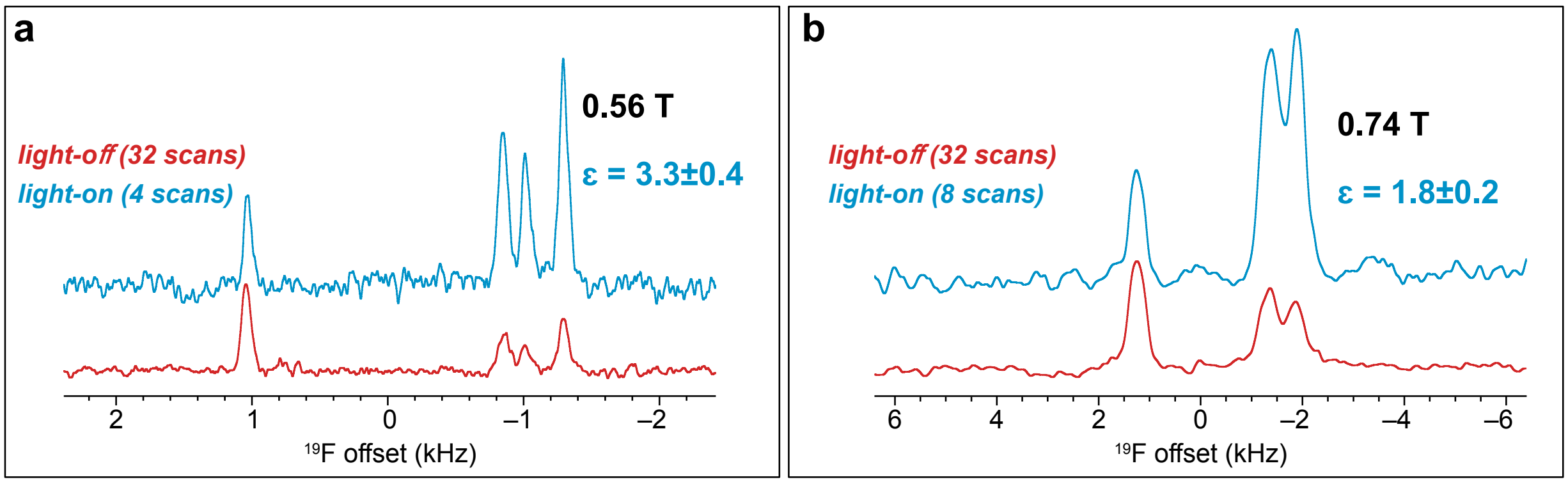

Figure S10. Liquid-state ^{19}F NMR spectra (blue: light-on, red: light-off) of a sample of 0.5 mM BDPA and 0.2 mM H_2TPP in perfluorotoluene/toluene-d_8 = 1/1 measured at a) 0.56 T and b) 0.74 T. The uncertainty on the enhancement factor has been estimated from the signal-to-noise ratio (snr) of the light-off spectrum as $\delta_\varepsilon = \varepsilon/\mathrm{SNR}_{\mathrm{light-off}}$.
